# Statement of Retraction: MicroRNA-30c-2-3p represses malignant progression of gastric adenocarcinoma cells via targeting ARHGAP11A

**DOI:** 10.1080/21655979.2026.2667583

**Published:** 2026-05-21

We, the Editors and Publisher of the journal *Bioengineered*, have retracted the following article from publication. 

Zheng, L., Cai, X., Song, J., Shi, H., Zhang, J., Ke, X., Li, H., and Chen, Y. (2022). MicroRNA-30c-2-3p represses malignant progression of gastric adenocarcinoma cells via targeting ARHGAP11A. *Bioengineered*, *13*(6), 14534–14544. https://doi.org/10.1080/21655979.2022.2090222 

Since publication, significant concerns have been raised about the integrity of the data and reported results in the article.

When approached for an explanation, the authors have not responded to our queries and so these serious concerns remain.  

As verifying the validity of published work is core to the integrity of the scholarly record, we are therefore retracting the article. The authors have been informed of this decision.

We have been informed in our decision-making by our policy on publishing ethics and integrity and COPE guidelines.  

The retracted article will remain online to maintain the scholarly record and will be digitally watermarked on each page as ‘Retracted’.  

